# Farmers’ perceptions and awareness of cattle feedlots as a climate-smart approach to enteric methane emissions

**DOI:** 10.1016/j.heliyon.2024.e39849

**Published:** 2024-10-25

**Authors:** Beautiful Isabel Mpofu, Mhlangabezi Slayi, Leocadia Zhou, Ishmael Festus Jaja

**Affiliations:** a Department of Livestock and Pasture Science, University of Fort Hare, Alice, 5700, South Africa; b Risk and Vulnerability Science Center, University of Fort Hare, Alice, 5700, South Africa

**Keywords:** Awareness, Cattle feedlots, Climate-smart, Enteric methane emission, Farmers’ perception

## Abstract

The perceptions of farmers regarding communal feedlots and their role in reducing enteric methane emissions have received limited attention in research. This study aims to examine farmers’ perceptions, assess awareness, and identify barriers to adopting feedlots as a climate-smart practice to enteric methane emissions in rural communities in the Eastern Cape Province, South Africa. 161 structured, paper-based questionnaires were distributed among smallholder cattle farmers around three feedlot locations. Most farmers had heard about feedlots (67.7 %); however, only a few (19.9 %) participated. The lack of knowledge about feedlot operations was the main factor influencing farmers’ participation in feedlots. Most farmers did not perceive feedlots as a climate-smart approach to reducing enteric methane output (53.8 %), with few sure (9.6 %) and others unsure (36.5 %). When asked about the reasons for their perceptions of feedlots as a climate-smart approach to mitigating methane, many farmers were unsure of the reasons (86.5 %), with the least believing it could be related to poor feed quality (1.9 %). The availability of financial resources and level of education were the key factors influencing farmers’ willingness to adopt feedlots as a climate-smart practice. These findings emphasise the importance of targeted educational workshops and support systems to enhance farmers’ awareness and promote the adoption of cattle feedlots as a climate-smart approach. The findings can also inform policy and practice to address perceived feedlot drawbacks, such as feed provision and technical assistance, and improve their successful implementation. However, further research is needed to explore farmers’ perceptions of cattle feedlots as a climate-smart practice.

## 1 Introduction

Livestock farming, particularly cattle farming, is a significant contributor to greenhouse gas emissions, with enteric methane being a potent greenhouse gas. Approximately 75 % of greenhouse gas emissions from the livestock sector can be attributed to cattle [1]. Climate change poses numerous uncertainties for livestock farming, including droughts, floods, heatwaves, and other natural disasters, which can harm animal health and productivity. Methane’s contribution to climate change exposes people, economic sectors, societies, and ecosystems to risks [2]. Farmers in communal areas are most vulnerable and susceptible to harm as they do not have adequate resources to deal with the impacts of climate change [3]. Increased mortality rates, heat stress, inadequate forage availability, and the outbreak of unpredictable diseases challenge the farmers as livestock heavily relies on natural resources for sustenance and survival. It is, therefore, crucial to explore and promote climate-smart practices that can mitigate these emissions and enhance the resilience of livestock farming.

Although cattle play a crucial role in the socio-economic aspects of rural communities, they are recognised as the primary contributors to methane production among animal species. Ruminants, including cattle, contribute approximately 16 % of global greenhouse gas emissions, with cattle alone accounting for 72.6 % of methane emissions from livestock [1,4]. Methane emissions are particularly prominent in cattle grazing on natural pastures, especially in communal areas. The methane problem arises because cattle in communal areas typically rely on highly lignified and low-digestible forage, leading to a significant loss of dietary energy through methane production during enteric fermentation [5]. However, feedlots offer a potential climate-smart practice by providing a controlled environment that can help mitigate animal methane losses, especially during winter [6].

A cattle feedlot is an agricultural activity designed to create a viable market for communal farmers by rapidly fattening their animals [7,8]. The communal feedlots program was implemented by the National Red Meat Development Programme (NRMDP) of the National Marketing Council (NAMC) to enhance smallholder cattle farmers’ participation in formal markets [9]. The initiative was to improve marketing channels of smallholder farmers’ agricultural products and increase their participation in feedlots. These feedlots offer several potential benefits, including reduced enteric methane emissions, improved weight gain in cattle, enhanced feed efficiency, and better utilisation of limited grazing resources [8]. Additionally, communal feedlots can provide opportunities for knowledge sharing, collective decision-making, and resource pooling among farmers. The Eastern Cape region, characterised by a substantial cattle population [10], exhibits the highest methane emissions profile from cattle, reaching 210 Gg [4]. The high methane emissions underscore the need to address the challenge of simultaneously increasing cattle productivity per animal while reducing methane emissions associated with cattle production.

In recent years, there has been growing interest in identifying and implementing climate-smart agricultural practices to mitigate the impact of climate change [11]. The adaptation measures identified include diversification, land use changes, adjustments in operational timing, and improved manure management, among others [12]. Despite employing these adaptation strategies, concerns about low productivity levels in communal areas have arisen. Consequently, farmers need help adopting and implementing these strategies due to ignorance and a lack of awareness regarding their importance in adapting to changing conditions [13].

Establishing cattle feedlots is one promising climate-smart practice to help farmers adapt and contribute to sustainable agricultural practices [8]. However, the success and widespread adoption of communal feedlots as a climate-smart practice depends on various factors, including farmers’ perceptions and awareness of their benefits. Farmers’ perceptions influence their decisions to adopt or reject new practices. Understanding farmers’ attitudes, beliefs, and knowledge regarding communal feedlots and their potential to mitigate the impact of climate change and enteric methane emissions is essential for effective policy development and successful implementation of such practices.

Rural communities in the Eastern Cape of South Africa play a vital role in livestock farming, making it essential to understand their perceptions and awareness of cattle feedlots [3]. This study is the first to explore smallholder farmers’ perceptions of feedlots as a climate-smart approach to methane reduction in the Eastern Cape Province’s Centane, Tsomo, and Middledrift rural areas. These areas are usually underresearched and often overlooked in the context of climate change and rural farmers’ perceptions of methane mitigation. Thus, examining farmers’ perceptions in these areas where feedlots are not widely adopted is essential for developing more sustainable agricultural practices that can improve livestock production and food security and enhance both environmental and economic outcomes. This research also provides a model for engaging smallholder farmers in feedlot practices, which could be replicated in other regions. Therefore, this study aims to examine farmers’ perceptions, assess awareness, and identify barriers to adopting feedlots as a climate-smart practice to enteric methane emissions in rural communities in the Eastern Cape Province, South Africa.

## 2 Materials and methods

### 2.1 Ethical approval

The study received ethical approval from the Inter-Faculty Research Ethics Committee (IFREC) at the University of Fort Hare, with the ethics number JAJ051SMPO01. Before participating in the study, a consent form agreement was provided for each interviewee to sign.

### 2.2 Study area

**Fig. 1:**
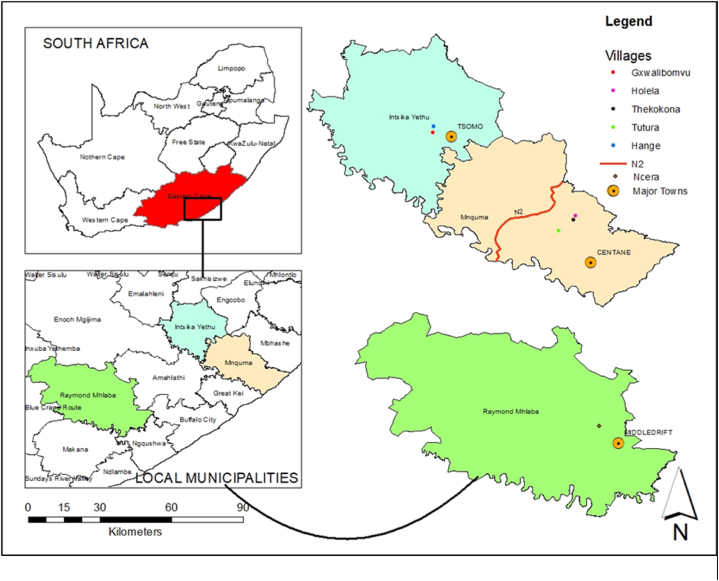
Map showing the study sites in Mnquma, Ntsikayethu, and Raymond Mhlaba Local Municipalities.

This study was conducted in the Centane, Tsomo, and Middledrift areas of the Eastern Cape Province, South Africa (Fig. 1). These towns were selected based on their proximity to the three communal feedlots implemented by the National Agricultural Marketing Council (NAMC). Under the program, structures simulating feedlots were erected, and NAMC donates subsidised commercial feed [9]. The feedlots were anonymously named F1 (Raymond Mhlaba) in Middledrift, F2 (Mnquma) in Centane, and F3 (Intsika Yethu) in Tsomo. Middledrift is located at 32.7° S and 27.03° E in Raymond Mhlaba municipality, Centane at 32.18° S and 28.02° E in Mnquma municipality, and Tsomo at 31.93° S and 27.64° E in Intsika Yethu municipality. Average minimum and maximum temperatures in these areas range from 9 °C to 22 °C, 8–25 °C and 20–25 °C respectively. These areas have most smallholder farmers from disadvantaged backgrounds who rely solely on government social grants and services for upkeep. Data collection for this study focused on villages within a 50-km radius of an existing feedlot. The villages included in the study were Gxwalibomvu, Hange, Tutura, Holela, Ncera, and Tekokona.

In the Raymond Mhlaba local municipality, the vegetation is predominantly Eastern Cape Thornveld and Amatole Montane Grassland, with notable grass species such as *Themeda tiandra, Pennisetum sphacelatum,* and *Ehrharta calycina* [14]. The municipality’s landscape features a flat, regular topography with shallow, rocky sand and mudstone soils with an average annual rainfall of 500 mm. The Mnquma Municipality climate varies from cool, humid, and subtropical along the coast to hot and sub‐arid further inland. The predominant vegetation in the municipality includes Bhisho Thornveld and Eastern Valley Bushveld savannas, with grasslands primarily found in the higher-lying northern section and annual rainfall ranging from 600 to 800 mm [15]. The Intsika Yethu local municipality experiences a climate characterised by increasing temperatures and less frequent but more intense rainfall, leading to a higher frequency and intensity of droughts and floods. The dominant vegetation type in the municipality is Tsomo grasslands, characterised by mudstone-dominant geology, making it favourable for crop cultivation due to its high nutrient content and water retention properties. The annual rainfall in the area ranges from 600 to 800 mm, with the highest amount typically occurring in March and the lowest in July.

### 2.3 Survey instrument

A survey was conducted on farmers’ perceptions of cattle feedlots as a climate-smart practice in reducing enteric methane emissions and weight loss. One hundred sixty-one (161) structured, paper-based questionnaires were distributed among communal cattle farmers in 3 feedlot locations: Raymond Mhlaba, Mnquma, and Intsika Yethu local municipalities. Ethics research confidentiality and informed consent were made available to the farmers to obtain their voluntary participation. The farmers were provided detailed information about the study, and their voluntary participation was sought through consent forms. Face-to-face interviews were conducted with the farmers to facilitate effective communication and understanding. The questionnaires were primarily written in English, but explanations and clarifications were provided in IsiXhosa when required. IsiXhosa is a dialect predominantly spoken in the Eastern Cape Province, where the study was conducted. A research team comprising five research assistants of Animal Science Master’s students and their supervisors was used for enumeration. This team worked collaboratively to ensure the smooth execution of the survey and to address any queries or concerns raised by the respondents.

### 2.4 Questionnaire design

The questions included in the questionnaire were developed based on the critical research questions to be answered by the study and benchmarked with previous studies [4,12,16]. It consisted of five sections, each serving a specific purpose. The first section focused on gathering demographic information. It included 12 questions related to the farmers’ experience in cattle farming, their age, gender, occupation, and whether cattle farming served as their primary source of income. The second section delved into communal cattle herd characteristics, featuring nine questions that explored the number of cattle owned by the farmers, whether they had ever sold or lost animals due to drought or climate shocks, and their reasons for selling animals. The third section addressed the impact of climate change on communal cattle, comprising seven questions. It aimed to capture changes noticed by the farmers that indicate a shifting climate and the strategies farmers employ to minimise production losses caused by climate change.

The fourth section focused on the farmers’ perceptions and participation in established communal feedlots. It consisted of 13 questions that examined farmers’ awareness of communal feedlots, their perceptions of sending cattle to feedlots, their willingness to participate in such programs, and the perceived benefits of sending cattle to feedlots. The fifth and final section of the questionnaire explored the farmers’ perceptions of feedlots as a mitigation approach to reduce enteric methane output. It included 16 questions that assessed farmers’ knowledge of enteric methane output, factors influencing the use of feedlots as an adaptation strategy to mitigate methane emissions, and the reasons behind cattle’s contribution to enteric methane output. In the demographic information and herd characteristics sections, farmers were provided with options to choose between “yes” or “no” or select an appropriate answer for each question or statement. The climate change and mitigation on communal cattle section also employed “yes” or “no” questions. Finally, in the section on farmers’ perceptions of enteric methane output, respondents were given the choice of “yes” or “no.” They were encouraged to provide their answers as necessary.

### 2.5 Data collection and sampling procedures

The data for this study was explicitly collected from farmers who owned cattle and resided within a 50-km radius of an existing feedlot. A snowball technique was employed to identify and select the farmers to be interviewed. The communal farmers who were ultimately sampled for the survey consisted of 161 individuals who expressed their willingness to participate after being provided with a comprehensive briefing on the purpose and objectives of the study. Among the collected questionnaires, 28 were obtained from farmers residing in the Raymond Mhlaba municipality, 53 from the Mnquma municipality, and 80 from the Intsika Yethu municipality. The identification process was based on the animals sent to the feedlots to distinguish between farmers who actively participated in feedlots and those who did not. This method allowed for a clear differentiation and understanding of the farmers’ involvement in feedlot activities.

### 2.6 Statistical analysis

The data collected from the structured questionnaires was meticulously entered into Microsoft Excel spreadsheets to ensure accurate record-keeping. Subsequently, the data was transferred to the Statistical Package for Social Sciences (SPSS) version 28 (SPSS Inc., Chicago, IL) for further analysis. Descriptive statistics, including frequencies, means, and percentages, were computed to provide an overview of the data. A significance level of 5 % was established as the criterion for statistical significance. The Kolmogorov-Smirnov normality test assessed whether the variables followed a normal distribution. However, the data did not exhibit a normal distribution, as evidenced by skewness and kurtosis values.

Consequently, the non-parametric method Chi-squared test (X2) was employed for the analysis. Upon analysing the data, it was found that the model-fitting information yielded significant results (p < 0.05), indicating that the model utilised was appropriate for the dataset at hand. The association between willingness to participate in feedlots and demographic variables was examined using the chi-squared test to assess the relationship between these factors. Furthermore, the relationship between feedlot locations and farmers’ perceptions regarding using the feedlots as a climate-smart approach to reduce enteric methane output was also investigated. This analysis aimed to determine any variations in perceptions across different feedlot locations. Regression analysis was also conducted to determine the key factors influencing the willingness to adopt feedlot practices as a climate-smart approach. Cronbach’s alpha was calculated to assess the internal consistency of the data gathered. The resulting value of 0.917 indicated a high level of internal consistency for the data, suggesting that the questions used in this section reliably measured the intended construct [17].

## 3 Results

### 3.1 Socio-economic characteristics and willingness of respondents to participate in feedlots

Table 1 displays the associations between participants’ willingness to engage in feedlots and various demographic variables, using data from 161 participants. Most respondents were willing to participate in feedlots (78.3 %), with the minority not interested (21.7 %). Most respondents willing to participate in feedlots were farmers older than 61 (36.6 %), while a minor proportion (6.2 %) fell within the 31–40 age range. Marital status was divided into four categories, with the highest proportion (45.3 %) being married individuals and the least being single individuals (21.1 %). In terms of gender, the majority of participants identified as male (54 %), while females accounted for 24.2 % of the sample. The highest participants’ education level was secondary education (27.3 %), followed by those with primary education (22.4 %). Occupation categories included unemployment (33.5 %) as the most common and business as the least common (1.2 %). When examining farming experience, most participants (60.2 %) had more than 11 years of experience in farming. However, there was no significant association (*p* > 0.05) between the willingness to participate in feedlots and all demographic variables (age, gender, marital status, education level, occupation).

**Table 1:** Association between willingness to participate in feedlots and demographic variables (n = 161).

Variables	Category	%		Total (%)		*X*^*2*^	*P-value*
		Yes	No	Yes	No		
Feedlot location	F1	24(14.9)	4(2.5)	**126**(78.3)	**35**(21.7)	1.4	0.49
	F2	42(26.1)	11(6.8)				
	F3	60(37.3)	20(12.4)				
Age (years)	1–30	20(12.4)	4(2.5)	**126**(78.3)	**35**(21.7)	1.6	0.81
	31–40	10(6.2)	4(2.5)				
	41–50	16(9.9)	3(1.9)				
	51–60	21(13)	5(3.1)				
	>61	59(36.6)	19(11.8)				
Marital status	Single	34(21.1)	7(4.3)	**126**(78.3)	**35**(21.7)	4.2	0.24
	Married	73(45.3)	20(12.4)				
	Widowed	18(11.2)	6(3.7)				
	Divorced	1(0.6)	2(1.2)				
Gender	Male	87(54)	22(13.7)	**126**(78.3)	**35**(21.7)	0.5	0.49
	Female	39(24.2)	13(8.1)				
Education level	None	7(4.3)	5(3.1)	**126**(78.3)	**35**(21.7)	5.5	0.24
	Primary	36(22.4)	13(8.1)				
	Secondary	44(27.3)	7(4.3)				
	Matric	21(13)	5(3.1)				
	Tertiary	18(11.2)	5(3.1)				
Occupation	Farming	14(8.7)	8(5)	**126**(78.3)	**35**(21.7)	4.7	0.32
	Employed	25(15.5)	4(2.5)				
	Pensioner	31(19.3)	10(6.2)				
	Unemployed	54(33.5)	12(7.5)				
	Business	2(1.2)	1(0.6)				
Farming experience (years)	1–5	17(10.6)	1(0.6)	**126**(78.3)	**35**(21.7)	5.3	0.07
	6–10	12(7.5)	1(0.6)				
	>11	97(60.2)	33(20.5)				
Is cattle farming the primary source of income?	No	110(68.3)	33(20.5)	**126**(78.3)	**35**(21.7)	1.3	0.25
	Yes	16(9.9)	2(1.2)				

Note: ∗Statistically significant at *p* < 0.05, *X*^*2*^: Chi-square, %: Percentage, F1: Raymond Mhlaba; F2: Mnquma; F3: Intsika Yethu, Total frequency of farmers willing or not willing to participate in feedlots are highlighted in bold.

### 3.2 Herd ownership characteristics and feedlot participation

Table 2 examines the association between communal cattle herd characteristics and feedlot locations based on data from 161 participants. The table provides information on different variables and their distribution across the feedlots, along with the results of the Chi-square tests for association. Most communal farmers owned cattle that ranged from 1 to 10 cattle per herd (65.8 %), with the least number of cattle found in the category of >41 (1.2 %). The number of cattle across feedlot categories was F1 (13.7 %), owning 1–10 cattle, while F2 had 23.6 % owning 11–20 cattle, and F3 had 28.6 % owning 21–30 cattle. Most farmers lost cattle due to drought and other climate shocks (59 %) and sold cattle for other reasons (50.3 %). Participants were asked about their reasons for selling cattle, including generating income, school fees, losing a loved one, and building and renovations. The most common reason was generating income (28.6 %) across all feedlots. The table also explores whether cattle were sold due to drought and other climate shocks, and the proportions varied across the feedlots. In F1, 14.9 % of participants reported selling cattle for climate-related reasons, while F2 had 29.2 %, and F3 had 40.4 %. However, the Chi-square test did not indicate a significant association (*p* > 0.05) between selling cattle due to climate shocks and feedlot locations. Overall, the analysis suggests that there were no significant associations (*p* > 0.05) between herd ownership characteristics (herd size, selling and losing cattle due to climate shocks) and the locations of the feedlots.

**Table 2:** Association between communal cattle herd characteristics and feedlot locations (n = 161).

Variables	F1(%)	F2(%)	F3(%)	Total (%)	*X*^*2*^	*P-value*
How many cattle do you own?						
1–10	22(13.7)	38(23.6)	46(28.6)	106(65.8)	9.4	0.31
11–20	5(3.1)	9(5.6)	21(13)	35(21.7)		
21–30	0(0)	5(3.1)	10(6.2)	15(9.3)		
31–40	0(0)	1(0.6)	2(1.2)	3(1.9)		
>41	1(0.6)	0(0)	1(0.6)	2(1.2)		
Total (%)	**28**(17.4)	**53**(32.9)	**80**(49.7)	**161**(100)		
Were cattle ever sold due to drought and other climate shocks?						
No	24(14.9)	47(29.2)	65(40.4)	136(84.5)	1.4	0.5
Yes	4(2.5)	6(3.7)	15(9.3)	25(15.5)		
Total (%)	**28**(17.4)	**53**(32.9)	**80**(49.7)	**161**(100)		
What were the reasons for selling cattle?						
Generate income	6(3.7)	11(6.8)	29(18)	46(28.6)	13.7	0.19
School fees	1(0.6)	2(1.2)	6(3.7)	9(5.6)		
Loss of a loved one	2(1.2)	1(0.6)	2(1.2)	5(3.1)		
Building/renovations	2(1.2)	3(1.9)	9(5.6)	14(8.7)		
Other	1(0.6)	1(0.6)	4(2.5)	6(3.7)		
N/A	16(9.9)	35(21.7)	30(18.6)	81(50.3)		
Total (%)	**28**(17.4)	**53**(32.9)	**80**(49.7)	**161**(100)		
Were cattle ever lost due to drought and other climate shocks?						
No	10(6.2)	25(15.5)	31(19.3)	66(41)	1.3	0.52
Yes	18(11.2)	28(17.4)	49(30.4)	95(59)		
Total (%)	**28**(17.4)	**53**(32.9)	**80**(49.7)	**161**(100)		

Note: ∗Statistically significant at *p* < 0.05, *X*^*2*^: Chi-square, %: Percentage; F1: Raymond Mhlaba; F2: Mnquma; F3: Intsika Yethu; Total frequency at each feedlot location is highlighted in bold.

### 3.3 Farmers’ perceptions and awareness of climate change impacts and adaptation strategies

Table 3 presents the association between farmers’ perceptions of climate change’s impact on cattle farming and feedlot locations based on a sample size of 161 participants. The first variable examines whether participants have heard of climate change. In feedlot F1, 5 % of participants had not heard of climate change, while 12 % had. In F2, 6.8 % were unaware, and 26.1 % were aware. In F3, 4.3 % were uninformed, and 45.3 % were informed. The Chi-square test showed a significant association (*p* < 0.05) between farmers’ awareness of climate change and feedlot locations. The second variable explores the changes participants have noticed in the environment, suggesting a changing climate. Across all feedlots, the most common changes reported were drought (63 %), heat stress (39 %), and dry pastures (39 %). The Chi-square tests indicated significant associations between these changes and feedlot locations, with varying degrees of significance (*p* < 0.05). The environmental changes led to farmers adapting to buying supplements and medication (65 %) and selling part of their cattle (19 %) to curb cattle production losses. The analysis reveals significant associations (*p* < 0.05) between farmers’ awareness of climate change, observed environmental changes, identified climate shocks, employed strategies, and feedlot locations. However, there was no significant association (*p* > 0.05) between feedlots, effective adaptation strategies, and reasons for not adapting.

**Table 3:** Association between farmers’ perceptions of climate change impact on cattle farming and feedlot locations (n = 161).

Variables	F1 (%) No Yes	F2 (%) No Yes	F3 (%) No Yes	Total (%) No Yes	*X*^*2*^	*P-value*
1. Have you ever heard of climate change	8(5) 20(12)	11(6.8) 42(26.1)	7(4.3) 73(45.3)	26(16) 135(84)	7.3	0.03∗
2. What changes have one noticed in the environment suggesting a changing climate?						
Dry winters	25(16) 3(2)	50(31) 3(2)	60(37) 20(12)	135(84) 26(16)	9.5	0.01∗
Drought	14(8.7) 14(8.7)	25(16) 28(17.4)	21(13) 59(37)	60(37) 101(63)	8.3	0.02∗
Heat stress	10(6.2) 18(11)	42(26) 11(6.8)	47(29) 33(20)	99(61) 62(39)	15	0.001∗
Dry pastures	10(6.2) 18(11)	38(24) 15(9.3)	51(32) 29(18)	99(61) 62(39)	10	0.01∗
Increased crop failure	16(9.9) 12(7.5)	46(29) 7(4.3)	59(37) 21(13)	121(75) 40(25)	8.8	0.01∗
3. Which climate shock gave more problems?						
Drought	13(8) 15(9.3)	14(8.7) 39(24.2)	8(5) 72(44.7)	35(22) 126(78)	18	0.001∗
Heatwaves	9(5.6) 19(11.8)	42(26) 11(6.8)	64(40) 16(9.9)	115(71) 46(29)		
Floods	23(14.3) 5(3.1)	51(31.7) 2(1.2)	58(36) 22(14)	132(82) 29(18)		
4. What was done to reduce production losses due to climate change?						
Sold part of cattle	14(8.7) 6(3.7)	39(24.2) 3(1.9)	51(32) 22(14)	104(65) 31(19)	8.6	0.01∗
Bought supplements and medication	1(0.6) 19(11.8)	9(5.6) 33(20.5)	20(12) 53(33)	30(19) 105(65)	4.6	0.1
Erected shades	20(12) 0(0)	38(23.6) 4(2.5)	62(39) 11(6.8)	120(75) 15(9)	3.8	0.15
Drilled a dam	19(11.8) 1(0.6)	37(23) 5(3.1)	64(40) 9(5.6)	120(75) 15(9)	0.9	0.64
Bought highly tolerant breeds	17(10.6) 3(1.9)	41(25.5) 1(0.6)	70(43.5) 3(1.9)	128(80) 7(4.3)	4.8	0.09
Sought government assistance	13(8) 7(4.3)	36(22) 6(3.7)	63(39) 10(6.2)	112(70) 23(14)	5.4	0.07
5. Which adaptation strategies were effective in reducing production losses						
Sold part of cattle	15(9.3) 5(3.1)	37(23) 5(3.1) 11(6.8) 31(19.3) 36(22.4) 6(3.7) 35(21.7) 7(4.3) 41(25.5) 1(0.6) 41(25.5) 1(0.6)	50(31) 23(14)	102(63) 33(21)	5.5	0.06
Bought supplements and medication	5(3.1) 15(9.3)		29(18) 44(27)	45(28) 90(56)	2.9	0.23
Erected shades	20(12) 0(0)		61(38) 12(7.5)	117(73) 18(11)	3.7	0.16
Drilled a dam	20(12) 0(0)		62(39) 11(6.8)	117(73) 18(11)	3.7	0.16
Bought highly tolerant breeds	19(11.8) 1(0.6)		66(41) 7(4.3)	126(78) 9(5.6)	2.3	0.31
Sought government assistance	17(10.6) 3(1.9)		65(40.4) 8(5)	123(76) 12(7)	3.5	0.17
6. If no strategies were employed, what were the reasons?						
Lack of financial resources	16(9.9)	27(16.7)	33(20.5)	76(47.2)	18	0.06
Lack of information	1(0.6)	7(4.3)	20(12.4)	28(17.4)		
Lack of awareness of climate change	2(1.2)	2(1.2)	10(6.2)	14(8.7)		
Multiple cattle ownership	0(0)	3(1.9)	4(2.5)	7(4.3)		
Other	1(0.6)	1(0.6)	4(2.5)	6(3.7)		
N/A	8(5)	13(8.1)	9(5.6)	30(18.6)		

Note: ∗Statistically significant at *p* < 0.05, *X*^*2*^: Chi-square, %: Percentage; F1: Raymond Mhlaba; F2: Mnquma; F3: Intsika Yethu.

### 3.4 Farmers’ engagement in feedlot activities within their local areas

Table 4 presents the association between farmers’ perceptions of using cattle feedlots and feedlot locations based on a sample size of 161 participants. Respondents were examined to check whether they had heard of feedlot or not. Most cattle farmers across all locations have heard about feedlots (71.4 %), and 28.6 % have never heard of them. 70.2 % of cattle farmers were aware of an existing feedlot in their area, and 29.8 % were unaware. The Chi-square test indicated a significant association (*p* < 0.05) between farmers’ awareness of feedlots and feedlot locations. The proportions show that most cattle farmers knew the feedlots; however, only a few participated in the feedlots (19.9 %). The farmers did not participate in feedlots for various reasons, with a lack of technical knowledge (41.6 %) being the most outstanding. There was a significant association (*p* < 0.05) between feedlot locations and reasons for not sending cattle to the feedlot and participation in feedlots. Most participants reported distances to the local feedlot between 2 and 10 km (46 %) and 11–20 km (16.8 %) across all feedlots. However, there was no significant association (*p* > 0.05) between feedlot locations and the estimated distance from the village to the feedlot.

**Table 4:** Association between farmers’ perceptions of using cattle feedlots and feedlot locations (n = 161).

Variables	Category	F1(%)	F2(%)	F3(%)	Total (%)	*X*^*2*^	*P-value*
Have you ever heard of a feedlot?	No	17(10.6)	23(14.3)	6(3.7)	46(28.6)	37.3	0.0001∗
	Yes	11(6.8)	30(18.6)	74(46)	115(71.4)		
	Total (%)	**28**(17.4)	**53**(32.9)	**80**(49.7)	**161**(100)		
Are you aware of the existence of a feedlot in the area?	No	19(11.8)	14(8.7)	4(2.5)	37(23)	61	0.0001∗
	Yes	9(5.6)	30(18.6)	74(46)	113(70.2)		
	Not sure	0(0)	9(5.6)	2(1.2)	11(6.8)		
	Total (%)	**28**(17.4)	**53**(32.9)	**80**(49.7)	**161**(100)		
What is the estimated distance from the village to the feedlot (km)	<1	0(0)	2(1.2)	6(3.7)	8(5)	9.5	0.30
	2–10	8(5)	16(9.9)	50(31.1)	74(46)		
	11–20	1(0.6)	9(5.6)	17(10.6)	27(16.8)		
	21–30	0(0)	1(0.6)	1(0.6)	2(1.2)		
	>31	0(0)	2(1.2)	0(0)	2(1.2)		
	Total (%)	**9**(5.6)	**30**(18.6)	**74**(46)	113(70.2)		
Have you ever sent cattle to the feedlot?	No	28(17.4)	51(31.7)	50(31.1)	129(80.1)	31.2	0.0001∗
	Yes	0(0)	2(1.2)	30(18.6)	32(19.9)		
	Total (%)	**28**(17.4)	**53**(32.9)	**80**(49.7)	**161**(100)		
What is the number of cattle sent to the feedlot?	1–10	0(0)	2(1.2)	28(17.4)	30(18.6)	0.1	0.71
	>21	0(0)	0(0)	2(1.2)	2(1.2)		
	Total (%)	**0**(0)	**2**(1.2)	**30**(18.6)	**32**(19.9)		
How often were animals sent to the feedlot?	During droughts		0(0)	8(5)	8(5)	0.7	0.4
	Any time of the year		2(1.2)	22(13.7)	24(14.9)		
	Total (%)		**2**(1.2)	**30**(18.6)	**32**(19.9)		
What could be the reason for stopping or never sending cattle to the feedlot?	Lack of technical knowledge	16(9.9)	35(21.7)	16(9.9)	67(41.6)	40.3	0.0001∗
	Stock theft	0(0)	0(0)	3(1.8)	3(1.8)		
	It is a personal choice	2(1.2)	6(3.7)	29(18)	37(23)		
	Unfavourable Policies/Risk	1(0.6)	6(3.7)	12(7.5)	19(11.8)		
	Others	9(5.6)	6(3.7)	20(12.4)	35(21.7)		
	Total (%)	**28**(17.4)	**53**(32.9)	**80**(49.7)	**161**(100)		
What changes would one like to see before participating in feedlots?	Transparent policies	2(1.2)	10(6.2)	28(17.4)	40(24.8)	21.1	0.007∗
	More awareness workshops	15(9.3)	30(18.6)	26(16.1)	71(44.1)		
	Improved infrastructure	3(1.8)	1(0.6)	2(1.2)	6(3.7)		
	None	8(5)	10(6.2)	17(10.6)	35(21.7)		
	Other	0(0)	2(1.2)	7(4.3)	9(5.6)		
	Total (%)	**28**(17.4)	**53**(32.9)	**80**(49.7)	161(100)		
Does sending cattle to feedlots assist in climate change mitigation	No	0(0)	8(5)	1(0.6)	9(5.6)	28.6	0.0001∗
	Yes	0(0)	1(0.6)	17(10.6)	18(11.2)		
	Not sure	28(17.4)	44(27.3)	62(38.5)	134(83.2)		
	Total (%)	**28**(17.4)	**53**(32.9)	**80**(49.7)	161(100)		

Note: ∗Statistically significant at *p* < 0.05, *X*^*2*^: Chi-square, %: Percentage; F1: Raymond Mhlaba; F2: Mnquma; F3: Intsika Yethu; Total frequency at each feedlot location is highlighted in bold.

In exploring the frequency of sending animals to the feedlot, cattle farmers were provided options for any time of the year (14.9 %) and during droughts (5 %). The Chi-square test did not indicate a significant association (*p* > 0.05) between the frequency of sending animals and feedlot locations. Upon investigation of the reasons for not sending cattle to the feedlot, the most reported reasons were a lack of technical knowledge (41.6 %), personal choice (23 %), and other reasons (21.7 %). The Chi-square test demonstrated a significant association (*p* < 0.05) between reasons for not sending cattle and feedlot locations. The most common changes farmers would like to see before participating in feedlots were more awareness workshops (44.1 %) and transparent policies (24.8 %), with the least being improved infrastructure (1.2 %). The Chi-square test indicated a significant association (*p* < 0.05) between desired changes and feedlot locations.

### 3.5 Farmers’ awareness and perception of feedlots benefits

Table 5 presents the association between participating farmers’ perceptions of feedlot benefits and feedlot locations based on a sample size of 32 participating farmers. The respondents were only from F2 and F3 feedlot locations, as the F1 feedlot location had no participating farmers. The first variable examines the perception of improved animal healthcare in cattle sent to the feedlots. In F2, no participants perceived improved animal healthcare, while 6.3 % did not. In F3, 63 % perceived improved healthcare, and 31 % did not. The Chi-square test did not indicate a significant association (*p* > 0.05) between this perception and feedlot locations. The second variable explores the perception of improved animal weight gain in feedlot cattle. In F2, 3.1 % perceived improved weight gain, and the same percentage did not. In F3, 75 % perceived improved weight gain, while 18.8 % did not. The Chi-square test did not indicate a significant association (*p* > 0.05) between this perception and feedlot locations. The third variable examines the perception of consistent feed and water availability in feedlots. In F2, none of the participants perceived consistent availability, while 6.3 % did not. In F3, 65.6 % perceived consistent availability, and 28 % did not. The Chi-square test indicated a significant association (*p* < 0.05) between this perception and feedlot locations.

**Table 5:** Association between participating farmers’ perceptions of feedlot benefits and awareness of feedlot locations (n = 32).

Variable	Feedlot awareness		Total(%)	*X*^*2*^	*P-value*
	F2(%)	F3(%)			
	Yes No	Yes No	Yes No		
1. There is improved animal healthcare in cattle sent to the feedlots	0(0) 2(6.3)	20(63) 10(31)	20(63) 12(37.5)	3.6	0.06
Total (%)			**32**(100)		
2. There is improved animal weight gain in feedlot cattle	1(3.1) 1(3.1)	24(75) 6(18.8)	25(78.1) 7(21.9)	1	0.32
Total (%)			**32**(100)		
3. There is consistent feed and water availability in feedlots	0(0) 2(6.3)	21(65.6) 9(28)	21(66) 11(34.4)	4.1	0.04∗
Total (%)			**32**(100)		
4. Feedlot provides an accessible market for communal farmers	0(0) 2(6.3)	22(68.8) 8(25)	22(68.8) 10(31)	4.7	0.03∗
Total (%)			**32**(100)		

Note: ∗Statistically significant at *p* < 0.05, *X*^*2*^: Chi-square, %: Percentage; F1: Raymond Mhlaba; F2: Mnquma; F3: Intsika Yethu; Text highlighted in bold is the total number of participating farmers in all feedlot locations.

The fourth variable assesses the perception that feedlots provide an accessible market for communal farmers. In F2, none of the participants perceived an accessible market, while 6.3 % did not. In F3, 68.8 % perceived an accessible market, and 25 % did not. The Chi-square test indicated a significant association (*p* < 0.05) between this perception and feedlot locations. The analysis shows a significant association between participating farmers’ perception of consistent feed and water availability in feedlots and the perception of feedlots providing an accessible market for communal farmers and feedlot locations. However, no significant associations were found between the perception of improved animal healthcare or improved animal weight gain and feedlot locations.

#### 3.5.1 Participating farmers’ responses to feedlot benefits

**Fig. 2:**
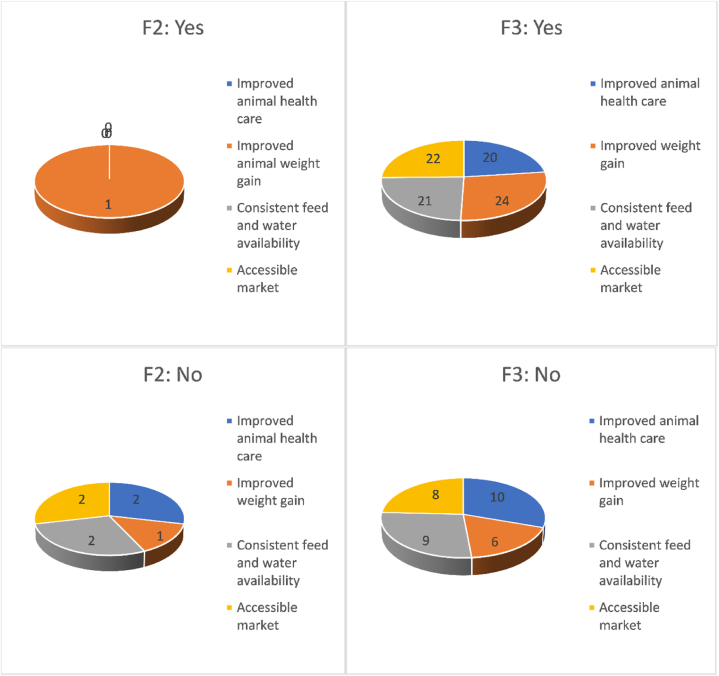
Pie chart of respondents on benefits of using communal feedlots (Note: F1: Not available; F2: Mnquma; F3: Intsika Yethu).

Fig. 2 illustrates the perceptions of participating farmers regarding the benefits of using communal feedlots. It displays the frequency of farmers from two feedlot locations, namely F2 and F3. No farmers from F1 participated; thus, their perceptions are not represented in the figure. Improved animal weight gain was the sole benefit perceived in the F2 feedlot location compared to F3. However, in F3, farmers held various perceptions regarding the benefits of feedlots. Some farmers from both F2 and F3 feedlot locations did not perceive improved weight gain, animal health care, accessible market, and consistent feed and water availability as advantages associated with using feedlots.

### 3.6 Farmers’ perceptions of cattle feedlots as a climate-smart approach to enteric methane output

Table 6 presents the association between farmers’ perceptions of feedlots as a climate-smart approach in reducing enteric methane and feedlot locations based on a sample size of 161 farmers. Across all feedlots, 67.7 % of farmers were unaware of methane, 24.8 % were unsure, and 7.5 % were aware. The Chi-square test indicated a significant association (*p* < 0.05) between awareness of methane and feedlot locations. Most farmers were unaware of the impacts of methane on the environment and livelihood (59.6 %) and were unsure about the contribution of different animals to enteric methane output (55.8 %). Farmers were aware of the impacts of methane on the environment, including global warming, droughts, and air pollution. The Chi-square test did not indicate a significant association (*p* > 0.05) between this awareness and feedlot locations. Most farmers did not perceive feedlots as a climate-smart approach to reducing enteric methane output (53.8 %), with few sure (9.6 %) and others unsure (36.5 %). However, no significant association (*p* > 0.05) exists between the perception of cattle feedlots as a climate-smart approach and feedlot locations. When asked about the reasons for their perceptions of feedlots as a climate-smart approach to mitigating methane, many farmers were unsure of the reasons (86.5), with the least perceiving that it could be due to poor feed quality (1.9 %). Few farmers perceived feedlots could reduce enteric methane output and weight loss in cattle (21.2 %). The chi-squared test results indicate a significant (*p* < 0.05) association between feedlot locations, knowledge of enteric methane output, and the contribution of animals to methane. However, there was no significant (*p* > 0.05) association between feedlot locations and multiple other variables (Table 6).

**Table 6:** Association between farmers’ perceptions of feedlots as a climate-smart approach to reduce enteric methane and feedlot locations (n = 161).

Variables	Category	F1(%)	F2(%)	F3(%)	Total (%)	*X*^*2*^	*P-value*
Have you ever heard of methane?	No	26(16.1)	36(22.4)	47(29.2)	109(67.7)	15.3	0.004∗
	Yes	1(0.6)	1(0.6)	10(6.2)	12(7.5)		
	Not sure	1(0.6)	16(9.9)	23(14.3)	40(24.8)		
	Total (%)	**28**(17.4)	**53**(32.9)	**80**(49.7)	**161**(100)		
Are you aware of the impact of methane on our environment and livelihoods?	No	2(3.8)	9(17.3)	20(38.5)	31(59.6)	8.7	0.07
	Yes	0(0)	1(1.9)	9(17.3)	10(19.2)		
	Not sure	0(0)	7(13.5)	4(7.7)	11(21.2)		
	Total (%)	**2**(3.8)	**17**(32.7)	**33**(63.5)	**52**(100)		
If yes, what impacts of methane on the environment are you aware of	Global warming	0(0)	0	2(3.8)	2(3.8)	6.5	0.59
	Droughts	0(0)	0	3(5.8)	3(5.8)		
	Air pollution	0(0)	0	4(7.7)	4(7.7)		
	All	0(0)	1(1.9)	2(3.8)	3(5.8)		
	None	2(3.8)	16(30.8)	22(42.3)	40(76.9)		
	Total (%)	**2**(3.8)	**17**(32.7)	**33**(63.5)	**52**(100)		
Can cattle feedlots be considered climate-smart in reducing enteric methane emissions from animals	No	1(1.9)	7(13.5)	20(38.5)	28(53.8)	7.4	0.11
	Yes	0(0)	0(0)	5(9.6)	5(9.6)		
	Not sure	1(1.9)	10(19.2)	8(15.4)	19(36.5)		
	Total (%)	**2**(3.8)	**17**(32.7)	**33**(63.5)	**52**(100)		
What could be the reason for the reduction of enteric methane by the feedlots?	Because they are kept in a small place	0(0)	0(0)	1(1.9)	1(1.9)	21.6	0.16
	Feed is already processed	0(0)	0(0)	1(1.9)	1(1.9)		
	The feed type is good	0(0)	0(0)	1(1.9)	1(1.9)		
	The cattle number remains the same	0(0)	0(0)	1(1.9)	1(1.9)		
	Poor feed quality	0(0)	0(0)	1(1.9)	1(1.9)		
	None	0(0)	0(0)	1(1.9)	1(1.9)		
	Not sure	2(3.8)	17(32.7)	27(51.9)	45(86.5)		
	Total (%)	**2**(3.8)	**17**(32.7)	**33**(63.5)	**52**(100)		
Using feedlots may help reduce enteric methane and improve weight gain.	No	1(1.9)	7(13.5)	19(36.5)	27(51.9)	3.9	0.42
	Yes	0(0)	3(5.8)	8(15.4)	11(21.2)		
	Not sure	1(1.9)	7(13.5)	6(11.5)	14(26.9)		
	Total (%)	**2**(3.8)	**17**(32.7)	**33**(63.5)	**52**(100)		
Reducing methane output could decrease the chance of drought.	No	1(1.9)	7(13.5)	20(38.5)	28(53.8)	3.6	0.47
	Yes	0(0)	1(1.9)	4(7.7)	5(9.6)		
	Not sure	1(1.9)	9(17.3)	9(17.3)	19(36.5)		
	Total (%)	**2**(3.8)	**17**(32.7)	**33**(63.5)	**52**(100)		
Feeding concentrates in feedlots helps reduce methane output.	No	1(1.9)	7(13.5)	22(42.3)	30(57.7)	4.4	0.35
	Yes	0(0)	1(1.9)	3(5.8)	4(7.7)		
	Not sure	1(1.9)	9(17.3)	8(15.4)	18(34.6)		
	Total (%)	**2**(3.8)	**17**(32.7)	**33**(63.5)	**52**(100)		
Sending or not sending cattle to the feedlot has no difference in weight gain.	No	1(1.9)	7(13.5)	23(44.2)	31(59.6)	5.5	0.24
	Yes	0(0)	1(1.9)	3(5.8)	4(7.7)		
	Not sure	1(1.9)	9(17.3)	7(13.5)	17(32.7)		
	Total (%)	**2**(3.8)	**17**(32.7)	**33**(63.5)	**52**(100)		

Note: ∗Statistically significant at *p* < 0.05, *X*^*2*^: Chi-square, %: Percentage; F1: Raymond Mhlaba; F2: Mnquma; F3: Intsika Yethu; Total frequency at each feedlot location is highlighted in bold; In cases where percentages do not add to 100, the respondents were not required to answer subsequent questions if they did not know about methane.

### 3.7 Factors influencing the willingness of farmers to adopt feedlot practises

A regression analysis of factors influencing farmers’ willingness to adopt feedlots as a climate-smart practice is presented in Table 7. The regression model was significant (F(8, 23) = 3.58, p = 0.008), indicating that the selected variables correlated with the willingness to adopt feedlot practices. The strongest predictors that influenced the adoption of feedlots by the farmers were the availability of financial services (β = 0.31, *p* = 0.001) and level of education (β = 0.48, *p* = 0.006). The lack of technical knowledge of the feedlot operations was negatively associated with the willingness to adopt feedlots (β = -0.43, *p* = 0.029). The more unfavourable the feedlot policies were, the fewer farmers were willing to utilise the feedlots (β = 0.041, p = 0.05). These are the critical barriers to farmers’ acceptability of feedlots, and these findings can inform policy and practice aimed at improving cattle production systems.

**Table 7:** Regression analysis of factors influencing willingness to adopt feedlots.

Variables	Coefficient (β)	Standard Error	t-Value	*p*-value
Intercept	0.707	0.204	3.466	0.002∗
Age of farmer	0.085	0.031	0.508	0.616
Level of education	0.479	0.030	2.994	0.006∗
Herd size	0.174	0.041	0.991	0.332
Availability of financial services	0.31	0.07	3.58	0.001∗
Access to feedlot information	−0.305	0.097	−1.647	0.113
Multiple cattle ownership	0.313	0.115	1.359	0.187
Lack of technical knowledge	−0.427	0.107	−2.335	0.029∗
Unfavourable feedlot policies	0.405	0.101	2.041	0.05∗
Feedlot awareness workshops	−0.115	0.027	−0.665	0.513

Note: ∗Statistically significant at *p* < 0.05, not statistically significant: *p* > 0.05.

## 4 Discussion

### 4.1 Socio-economic characteristics and willingness of participants

The study findings revealed that most respondents willing to participate in feedlots were male farmers aged 61. This observation aligns with previous researchers who reported that many smallholder farmers in various contexts were older individuals [[18], [20]]. This trend suggests a declining interest among the younger generation in pursuing agricultural practices. Furthermore, the higher representation of male farmers than female farmers indicates the persistence of gender stereotypes within the farming community. Agriculture has traditionally been considered a male-dominated field, and the imbalance in gender representation reflects the existing gender biases. Addressing these disparities and promoting equal participation and opportunities for both male and female farmers is crucial. However, it is essential to note that the demographic variables, including age and gender, did not significantly affect the willingness to participate in feedlots. This finding suggests that these factors did not directly influence the farmers’ inclination to communal feedlot practices. Instead, other considerations and factors may have played a more prominent role in shaping their decisions.

The smallholder communal farmers had a positive inclination towards participating in feedlots. They expressed their desire to implement specific changes before their active involvement. The critical changes identified by the farmers included increased awareness workshops and transparent pricing mechanisms. The communal farmers indicated they would be more willing to participate if provided with information about the operations and benefits of feedlots. They emphasised the need for workshops to equip them with knowledge about the purpose and significance of feedlots in their agricultural practices. One major constraint communal farmers reported was the lack of information regarding communal feeding schemes and pricing structures. Previous research highlighted this constraint [22] as a significant market barrier for communal farmers. Addressing this information gap and establishing transparent pricing systems would enhance communal farmers’ participation in feedlot initiatives.

### 4.2 Herd ownership characteristics and feedlot participation

The study findings revealed that the number of cattle owned by farmers influenced their decision to participate in feedlots. A significant proportion of communal farmers owned fewer than ten cattle, indicating a relatively low population in communal areas. The results are consistent with previous research, which reported herd sizes ranging from one to a maximum of twenty-one cattle farmers, with an average of 10 heads per farmer [19,23]. However, these findings contrast with other studies where higher cattle population numbers were observed [24,25]. The disparity in findings may be attributed to the impact of drought, resulting in significant cattle mortality and leaving farmers with limited or no cattle. Consequently, farmers had fewer cattle available to allocate to feedlots, leading to lower participation rates.

The primary reasons for cattle production among smallholder farmers also influenced their decisions to participate in feedlots. Most communal farmers kept and sold cattle primarily for income generation, with a minor consideration given to funeral proceedings. These findings align with similar studies where farmers emphasised cattle as a source of income and family support [19]. Selling cattle helped the communal farmers cater for school fees and food. Selling cattle provided financial means for communal farmers to cover expenses such as school fees and food. As communal farmers typically sold cattle for specific purposes, they were reluctant to send them to feedlots. Farmers preferred to hold onto their cattle, anticipating selling them when there was a domestic need [26]. Cattle were perceived as a means of livelihood and wealth, leading to hesitance in selling them.

It is worth noting that communal farmers often considered older animals more valuable despite market trends suggesting the opposite. This knowledge of cattle pricing at markets significantly influenced farmers’ perceptions and decisions regarding participation in feedlots. When communal farmers sold their cattle through feedlots, the poor condition of the animals, including health issues and advanced age, often resulted in lower grading per kilogram [6]. The reduced value of aged animals in the market further discouraged farmers from selling their cattle. Consequently, these animals continued to age and died, representing a loss for the farmers. The findings highlight the complex interplay between cattle ownership, farmers’ economic needs, market dynamics, and the reluctance to sell aged animals. Understanding these factors is crucial for designing interventions that address the specific challenges faced by communal farmers and promote their participation in feedlot initiatives. Strategies that provide support in improving cattle health, marketing knowledge, and alternative income-generation opportunities may help alleviate the constraints faced by communal farmers and enhance their engagement in climate-smart practices.

### 4.3 Farmers’ perceptions and awareness of climate change impacts and adaptation strategies

The study findings revealed that farmers in the Eastern Cape Province were highly aware of climate issues and held explicit opinions on environmental changes resulting from climate change. These results align with previous studies that examined livestock farmers’ perceptions of climate change, indicating that farmers had varied perceptions of climate change [11,12,27,28]. Climate change was primarily perceived in terms of droughts, floods, heat stress, and increased crop failures occurring unpredictably throughout the year, leading to disasters. These perceptions indicate that the farmers in the region were aware of climate change.

The results also shed light on farmers’ perceptions of strategies to mitigate cattle production losses due to climate change in each feedlot area. These findings are consistent with several studies that explored farmers’ perceptions of climate change and adaptation strategies, revealing diverse perceptions among farmers [12,21,29,30]. Farmers residing around the F2 feedlot area perceived relocating to areas with greener pastures as an effective strategy. They also expressed the need for financial support to construct shades and protect animals from extreme weather conditions. Furthermore, farmers acknowledged the importance of seasonal feed supplements and medication to mitigate production losses caused by climate change, although they highlighted affordability challenges in purchasing them.

Smallholder farmers expressed the need for more government assistance, particularly in dipping and vaccination programs that were no longer provided. Their perception of government assistance aligns with the negative perceptions found in the study, where farmers had reservations about the effectiveness of government assistance in coping with climate change [31]. Farmers mentioned that seeking government aid during climatic challenges often proved unhelpful, as the government primarily assisted the unemployed, leaving employed farmers excluded from potential benefits. Although the government provided limited Lucerne supplements, they were insufficient to meet the needs of the entire village, forcing farmers to purchase expensive supplements and medication. As communal farmers relied heavily on social grant money for their livelihoods, additional financial resources were required to cover the costs of cattle supplements.

Farmers in the villages surrounding the F3 feedlot location perceived strategies such as erecting shades and drilling dams as ineffective in combating production losses. The lack of infrastructure, including dams and sheds, posed challenges for farmers in mitigating production losses caused by climate change. Therefore, adopting strategies such as dam construction and shed erection becomes essential to alleviate the impacts of drought on livestock and livelihoods. Adaptation strategies play a crucial role in enhancing the resilience of farmers and reducing livestock production vulnerability to climate shocks [32,33]. Assessing communal farmers’ perceptions of climate change resilience is vital for effectively planning and improving climate-smart strategies.

Farmers in the villages surrounding the F1 feedlot location adopted a strategy of selling cattle to mitigate production losses resulting from climate change. The proceeds from selling part of their stock were used to purchase medication for the remaining herd. These smallholder farmers had limited knowledge of available animal medicines, and due to financial constraints, they rarely purchased supplements such as Lucerne. The significant association between selling cattle as an adaptation strategy to reduce production losses and feedlot location suggests that the feedlot location influenced farmers’ decisions to sell cattle. Farmers perceived selling part of their cattle as an effective strategy to minimise production losses.

### 4.4 Farmers’ engagement in feedlot activities within their local areas

The study findings revealed that most communal farmers knew of feedlots in their area and were aware of their presence. However, there was a significant association between farmers’ perceptions of sending cattle to the feedlot and the location of the feedlot, indicating that the feedlot location influenced farmers’ participation in communal feedlots. Communal farmers may have refrained from participating in feedlots for various reasons. While they knew the feedlot’s existence, they lacked information about its operations. This knowledge gap poses a challenge that must be addressed to enhance communal cattle productivity per head, as feedlot operations are an integral part of cattle production.

These findings are consistent with studies that identified significant constraints in communal cattle farmers’ production, such as lack of knowledge, financial resources, limited management skills, and disease control [19,26]. Therefore, there is a clear need to provide knowledge and information about feedlot operations to smallholder communal farmers. The lack of understanding regarding feedlot operations led communal farmers to perceive them as belonging to the chiefs or other individuals rather than being accessible to them. Consequently, farmers hesitated to send their cattle to the feedlot as they did not consider themselves potential participants. Raising awareness among farmers about the purpose and benefits of existing feedlots is crucial, helping them recognise that these facilities are also meant for their use.

The significant association between reasons for non-participation in feedlots and the feedlot location suggests that farmers’ decision not to participate may be influenced by factors specific to the feedlot itself. Unfavourable pricing by the feedlot was one of the reasons farmers cited for not participating in the F3 feedlot location. Farmers expressed dissatisfaction with prices favouring the feedlot more than the farmers themselves. Unfavourable pricing practices, where the feedlot set prices as they wished, deterred farmers from sending their cattle to the feedlot. A lack of market price information is a potential barrier that prevents communal smallholder farmers from utilising available markets [34].

Some farmers chose not to participate in feedlots, and these choices were influenced by their ability to afford feed supplements and animal medication and their perception of communal feedlots. To some farmers, adequate rainfall provided sufficient pastures for their cattle, eliminating the need for feed supplementation. Additionally, some farmers preferred selling their cattle through the informal or traditional market due to mistrust and concerns about corruption within the feedlot system [19,34]. These factors contributed to farmers selling their cattle through alternative channels, leading to a limited number of cattle available for participation in feedlots.

#### 4.4.1 Barriers and facilitators influencing farmer’s participation in feedlots

Communal feedlots are a viable climate-smart approach for improving cattle weight performances and reducing methane emissions. However, several challenges hinder the successful implementation of communal feedlots. These challenges include the need for adequate infrastructure, availability of resources, and transparency in feedlot operations. Communal farmers perceived poor infrastructure as a reason for their disengagement from feedlots. Inadequate infrastructure contributes to stock theft, fostering distrust toward feedlots among communal farmers [19]. Proper feedlot structures are crucial for communal farmers. They provide improved animal nutrition and health, enhanced weight gain, and knowledge sharing, contributing to cattle productivity in communal areas and farmers’ livelihoods. Communal feedlots must prioritise and ensure feed availability for cattle, especially during drought and dry seasons [35]. Since most communal feedlots are community-owned and rely on the NAMC program for feed supply [6], the inconsistency of the program becomes a significant problem for the progress and sustainability of these feedlots.

The long distance from the feedlot also deterred farmers from participating in feedlots. Farmers at least 2 km from the feedlot faced challenges transporting their cattle due to the distance and lack of funds for hiring vehicles. The considerable distance between farmers and the feedlot, particularly in the F1 location, acted as a barrier to the participation of communal farmers in formal markets [34,36,37]. Smallholder farmers often prefer on-farm cattle sales to avoid the high transactional costs of transporting cattle over long distances [22]. Additionally, some farmers did not see the need to send their cattle to feedlots as they kept them for subsistence farming.

Lack of financial resources was another significant reason communal farmers could not participate in feedlots. Feedlot managers required farmers to bring tagged animals, and farmers needed additional funds to purchase the tags. These findings align with previous research [9], where communal farmers expressed unwillingness to bear additional costs for branding and transportation, citing these as the primary barriers to their participation in feedlots. Since most communal farmers rely solely on social grants for their livelihoods and are unemployed [38], the funds from social grants were insufficient to cover their food expenses while purchasing the necessary tags for feedlots. Food insecurity creates the need for a ‘climate-smart system’ that is more resistant to the impacts of climate change on food security. The low participation observed in this study contrasts with other findings with a higher feedlot engagement [8]. The participation in this study can be primarily attributed to the key factors that influenced the adoption of feedlots as a climate-smart practice, including lack of financial resources and the level of education. Farmers with a higher level of education are more likely to be willing to participate in feedlots and have a greater awareness of the benefits and challenges involved [39]. These findings can inform the policy to improve cattle production through feedlot systems in smallholder areas.

### 4.5 Farmers’ awareness and perception of feedlot benefits

The participating smallholder farmers perceived the most significant benefit of communal feedlots to be the improvement in the weight performance of cattle sent to the feedlots. This finding aligns with previous research indicating that cattle sold through communal feedlots tend to weigh more than those not benefiting from such schemes [40,41]. Therefore, communal feedlots positively impact the weight performance of cattle. This improved weight is achieved by providing a balanced diet that meets the nutritional needs of the animals, enabling them to reach their full growth potential and resulting in heavier weights. Notably, there was no significant association between improved weight gain and feedlot locations, suggesting that the specific location of the communal feedlots did not influence farmers’ perception of improved weight gain.

However, a few participating farmers disagreed with the improvement in weight gain in the F2 and F3 feedlot locations. These findings contradict those of other researchers who have observed significantly higher weights in cattle sold through communal feedlots due to the supplementary communalised feeding regime implemented [9,40,41]. The contradiction may be attributed to feed shortages experienced at the feedlots, as highlighted by some participating farmers. Insufficient feed availability can lead to undernourished animals, resulting in poor weight gain and overall health. Addressing feed shortages promptly and adjusting the diet to ensure the animals receive optimal nutrition is crucial. Additionally, farmers may consider implementing regular weight monitoring of cattle in feedlots to ensure they are achieving their desired weight gain.

Smallholder farmers also perceived that cattle feedlots ensured consistent feed and water availability and provided an accessible market, encouraging them to send more cattle to the feedlots. The significant association between these variables and feedlot locations indicates that the specific location of the feedlot influenced farmers’ perception of it as an accessible market. The readily accessible market enabled farmers to sell their cattle anytime, generating income. Feedlots play a crucial role in enhancing market access for farmers in communal areas, allowing them to capitalise on favourable market conditions and generate income. By raising animals in a controlled environment, farmers can produce livestock of consistent quality, which buyers and processors highly value. Furthermore, farmers perceived that feedlots contributed to youth employment and provided opportunities for the local community. This employment creation adds to the overall significance of livestock production for human livelihood, which has been relatively scarce in the literature [42].

### 4.6 Farmers’ perception of cattle feedlots as a climate-smart approach to enteric methane emissions

The study revealed that most communal smallholder farmers had never heard of enteric methane output, and the feedlot location significantly influenced this lack of awareness. These findings align with existing literature indicating that farmers generally lack knowledge about enteric methane emissions [11]. Specifically, the F3 feedlot location had the highest number of farmers who had never heard of enteric methane output compared to the F1 and F2 locations. Communal smallholder farmers often rely on traditional farming practices passed down through generations, and these practices may not prioritise or emphasise environmental sustainability or consider the specific issue of enteric methane emissions. As a result, farmers may not actively seek information on this topic or prioritise its integration into their farming methods.

When asked about the contribution of feedlots to enteric methane output, most farmers from the F3 location perceived that feedlots produced more methane than farmers from F1 and F2. These farmers had previously participated in feedlots but stopped due to the perceived terrible taste of meat from feedlot cattle compared to non-feedlot cattle. They believed the unpleasant taste resulted from increased methane production in feedlot cattle. These findings are consistent with studies that have observed increased methane production in feedlot animals [44]. However, the farmers’ perceptions contradict the results of many other researchers who have found that feedlot cattle do not necessarily produce more enteric methane than non-feedlot cattle [[11], [27],]. The reason for this discrepancy may be the farmers’ belief that cattle sent to the feedlot produce more methane due to the low-quality feeds provided to the animals in the feedlot.

Interestingly, most communal farmers demonstrated awareness of the contribution of animals to enteric methane output, although they were not confident in their knowledge of its impact on animal performance [11,17]. However, only a few farmers perceived that feedlots could be used as a climate-smart approach to reduce enteric methane output and weight loss in cattle. Their belief was based on factors such as the availability of high-quality feed and the compact area in which the animals are kept. High-quality feed can provide the necessary nutrition for the animals, promoting their health and potentially reducing methane output [45,46]. These farmers knew how to mitigate enteric methane emissions through feedlot practices.

### 4.7 Feedlots as a support mechanism for enhancing enteric methane reduction: insights and implications

While facing the challenge of reducing the environmental impact of enteric methane emissions, communal farmers need to sustain their livelihoods through improved livestock production practices. Communal feedlots provide farmers a viable solution to increase cattle productivity per head. Most communal farmers did not participate in feedlots, and the few participating farmers surveyed perceived an improved weight gain in feedlot cattle, demonstrating this solution’s effectiveness. Therefore, communal feedlots can be a successful climate-smart approach for farmers looking to reduce weight loss in their cattle, provided all farmers are given training or information on feedlots. In addition, feedlots can be used at any time of the year, including periods of drought, to help reduce weight loss in cattle.

Most farmers did not know about enteric methane and its environmental impact. Educating farmers on the impact of enteric methane output on animal performance and the environment, as well as the role of communal feedlots in reducing methane output, could be beneficial in helping farmers make informed decisions on their farming practices. Additionally, providing resources and support, such as awareness workshops for farmers, could help bridge the knowledge gap and ensure that all farmers can access enteric methane information. Some farmers perceived feedlots to increase enteric methane output instead of reducing it. Hence, more evidence is needed to account for farmers’ perceptions of the reduction of enteric methane output by feedlot practices; however, improvement may be accomplished with good management and proper feedlot structures.

### 4.8 Limitations

The study focused on a specific geographic area, which may limit the applicability of the findings to other regions or contexts. Different regions may have unique socio-economic and environmental factors influencing farmers’ perceptions and awareness of communal feedlots. It is essential to consider this limitation when interpreting the study’s findings and recognise the need for further research to address them and provide a more robust understanding of farmers’ perceptions and awareness of communal feedlots as a climate-smart practice.

## 5 Conclusion

Most farmers were aware of cattle feedlots, and their perceptions of feedlots as a climate-smart approach to reducing enteric methane output varied. Some farmers believed that there was only a slight reduction or no reduction in methane output in feedlots and chose not to adopt feedlots. Their decision was influenced by their perception that the increased enteric methane output negatively affected the taste of the meat. The farmers had the perception that the region is more susceptible to drought, dry pastures, and heat stress, possibly due to the release of enteric methane, which contributes to global warming and the subsequent rise in temperatures. These climate-related challenges pose difficulties for farmers and negatively affect the environment.

On the other hand, some farmers perceived that weight loss was reduced in cattle sent to the feedlots, as evidenced by improved weight performances. This evidence suggests that communal feedlots could serve as a climate-smart approach to mitigate weight loss in cattle. However, considering the varying perceptions of enteric methane reduction, further research is needed to explore and understand the farmers’ perspectives on using communal feedlots as a climate-smart approach to enteric methane output and weight loss in cattle. Such research would provide valuable insights into the potential benefits and challenges of implementing communal feedlots as a climate-smart practice in the study area.

### 5.1 Policy recommendations

#### 5.1.1 Promoting awareness and educational programs

This research identified a gap in awareness of the purposes of the feedlot structures in the rural communities of the Eastern Cape Province. The National Red Meat Development Programme (NRMDP) of the National Marketing Council (NAMC) implemented the communal feedlots program. It is recommended that NAMC and agricultural extension services develop and implement targeted awareness and education programs to inform farmers about the benefits of feedlots in reducing methane emissions and improving cattle productivity.

#### 5.1.2 Financial and feed assistance

The farmers acknowledged they have benefited from the feed distribution by NAMC and the Department of Rural Development and Agrarian Reform (DRDAR). However, these feeds were insufficient as DRDAR only sponsored the farmers for a short period. It is recommended that DRDAR policymakers help provide farmers with unlimited access to feed and create financial incentives (subsidies, grants, low-interest loans).

#### 5.1.3 Improved infrastructure

One of the barriers to adopting feedlots identified in the study was the lack of infrastructure, which increased the distance from the village to the feedlots. Government agencies and NAMC could help establish more feedlot structures in rural communities and develop policies that promote their adoption. Easy access to these will encourage farmers to adopt feedlots. Proper feedlot structures that promote animal welfare will help smallholder farmers foster collaborations between different stakeholders, including researchers, policymakers, and private companies, thus improving overall livestock production. Policymakers establish monitoring and evaluation frameworks to assess the effectiveness of feedlot adoption and its impact on methane reduction and farmer livelihoods. This could help refine policies and ensure continuous improvement.

## CRediT authorship contribution statement

**Beautiful Isabel Mpofu:** Writing – review & editing, Writing – original draft, Software, Project administration, Methodology, Investigation, Formal analysis, Data curation, Conceptualization. **Mhlangabezi Slayi:** Writing – review & editing, Supervision, Resources, Data curation, Conceptualization. **Leocadia Zhou:** Supervision, Resources, Project administration. **Ishmael Festus Jaja:** Writing – review & editing, Supervision, Resources, Data curation, Conceptualization.

## Ethics statement

The study received ethical approval from the Inter-Faculty Research Ethics Committee (IFREC) at the University of Fort Hare, with the ethics number JAJ051SMPO01.

## Data availability

Data is freely available upon request from the corresponding author(s).

## Funding

We acknowledge the financial support from the South African Society of Animal Science (SASAS) and National Research Foundation (Grant number: PMDS22061422419). Gratitude is also given to the Risk and Vulnerability Science Centre (Grant number: TS64) and the Department of Livestock and Pasture Science for help with the research logistics.

## Declaration of competing interest

The authors declare that they have no known competing financial interests or personal relationships that could have appeared to influence the work reported in this paper.
