# Supplementary material for: Farmers’ perceptions and awareness of cattle feedlots as a climate-smart approach to enteric methane emissions
Source: Heliyon. 2024 Oct 25;10(21):e39849. doi: 10.1016/j.heliyon.2024.e39849 (PMC11550634; doi:10.1016/j.heliyon.2024.e39849)
Supplement: Multimedia component 1 [file mmc1.docx]

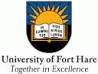


**Research questionnaire for farmers’ perceptions and awareness of feedlots as a climate-smart approach to reduce enteric methane and weight loss in cattle**

Dear participant,

My name is Beautiful Isabel Mpofu, and I am an MSc student studying animal science at the University of Fort Hare. I am researching using feedlots as an adaptation approach to lower enteric methane output and weight loss in cattle in the Eastern Cape Province, South Africa (Ethics number: **JAJ051SMPO01**). The study intends to document the acceptability of communal feeding kraals as an adaptation approach to ongoing climate crises and the impact of enteric methane emissions and weight loss in rural communities. The information generated in the current study will only be used for academic purposes and provide insights into some of the constraints or concerns resulting in the low participation of farmers in communal cattle feeding schemes in the area.

The participant may not participate in this research as it is not by force. It would, however, be appreciated if one shared their ideas and input with us. The information will remain confidential as we will not record names anywhere on the questionnaire. Therefore, the answers cannot be traced back to the participants. Further explanation will be provided to the participant, where possible.

Informed Consent

I agree to participate in research regarding communal feedlots as an adaptation approach to reduce enteric methane impacts and weight loss in cattle in the Eastern Cape Province, South Africa. I understand that participation is voluntary and by choice, without being forced in any way to do so. I understand that this consent form will not be linked to the questionnaire and that answers will remain confidential.

………………………… ……………………… Signature of participant Date

# Demographic information

| Municipality |  | | | | | Village | | |  | | | | | | |
| --- | --- | --- | --- | --- | --- | --- | --- | --- | --- | --- | --- | --- | --- | --- | --- |
| Gender | Male | | | | | Female | | |  | | | | | | |
| Age | 1-30 |  | 31-40 | |  | 41-50 | |  | 51-60 | |  | >61 | | |  |
| Marital Status | Single |  | Married | |  | Widowed | |  | Divorced | |  | Other | | |  |
| Education | None |  | Primary | |  | Secondary | |  | Matric | |  | Tertiary | | |  |
| Occupation | Farming |  | Employed | |  | Pensioner | |  | Unemployed | |  | Business | | |  |
| Is cattle farming your primary source of income? | | | | | Yes | |  | | | No |  | | | | |
| If not, which other farming streams do you get income from? | | | | | Smallstock | |  | | | Food Crops |  | | | | |
| Years of experience in cattle farming? | | | | | 1-5 | |  | | | 6-10 |  | | >11 |  | |
| Apart from cattle, do you own any other livestock species? | | | | | Yes | |  | | | No |  | |  |  | |
| If yes, how many are from these groups? | | | | Goats | Sheep | |  | | | Chicken |  | | Pigs |  | |

**B. Herd characteristics**

| How many cattle do you have? | 1-10 |  | 11-20 |  | 21-30 |  | 31-40 | |  | <41 | |  |
| --- | --- | --- | --- | --- | --- | --- | --- | --- | --- | --- | --- | --- |
| What number of cattle groups do you have? | Bull |  | Cows |  | Heifer |  | Steers | |  | Calves | |  |
| In the last 3 years, how many did you sell based on cattle groups? | Bull |  | Cows |  | Heifer |  | Steers | |  | Calves | |  |
| At what price do you usually sell? | Bull |  | Cows |  | Heifer |  | Steers | |  | Calves | |  |
| What was the reason for selling these animals? | Generate income |  | School fees |  | Loss of a loved one |  | Building or renovations | |  | Other | |  |
| Did you ever sell your animals due to droughts and other climate shocks? | Yes |  | No |  | | | | | | | | |
| Did you lose animals due to drought and other climate shock? | Yes |  | No |  | | | | | | | | |
| How many animals died due to droughts and other climate shocks? | Pregnant cows |  | Lactating cows |  | Bull |  | Steers |  | Heifers |  | Calves |  |
| Estimate financial losses for these animals | Pregnant cows |  | Lactating cows |  | Bull |  | Steers |  | Heifers |  | Calves |  |

1. **Climate change impact and mitigation on communal cattle**

| Have you ever heard of climate change? | **Yes** |  | | | **No** |  | | |
| --- | --- | --- | --- | --- | --- | --- | --- | --- |
| What changes have you noticed in your environment that suggest a changing climate? | Winters are extremely dry | High incidences of drought | Heat stress | Dry pastures | Increased crop failure | N/A | | |
| Which climate shock gave you more problems? | Drought |  | Heatwaves |  | Floods |  | | |
| Which climate extremes affected your cattle farming the most? | Vegetation loss |  | Disease outbreaks |  | Weight loss |  | Increase death rate and abortion. |  |
| How did you try to avoid these production losses due to climate shocks? | Sell a portion of the stock | Buy supplements and medication | Erected shades | Drilled a dam | Bought highly tolerant breeds | Seek government assistance | | Other |
|  |  |  |  |  |  |  | |  |
| Which strategies were effective in reducing production losses? Please select the most appropriate. | Selling a portion of the stock | Buy supplements and medication | Erected shades | Drilled a dam | Bought highly tolerant breeds | Seek government assistance | | Other |
|  |  |  |  |  |  |  | |  |
| What could be the reason for not adopting any of these strategies? | Lack of money | Lack of information | | Lack of awareness of climate change | | Multiple cattle ownership | | Other |

1. **Farmer’s knowledge and participation in established communal feeding kraals**

| Have you ever heard of feedlots? | Yes |  | No |  | | | | | | | | | | | | | |
| --- | --- | --- | --- | --- | --- | --- | --- | --- | --- | --- | --- | --- | --- | --- | --- | --- | --- |
| Are you aware of the existence of a feedlot in your area? | Yes |  | No |  | | | | | | | | | | | | | |
| What is the estimated distance to the feedlot? (km) | <1km |  | 2-10km |  | 11-20 |  | 21-30 | |  | | >31km | | | | |  | |
| Have you ever sent animals to the feedlot? | Yes |  | No |  | If yes, how many were sent? | 1-10 | | 11-20 | |  | | | >21 | | |  | |
|  |  |  |  |  | The number sent according to group | Bulls | | Cows | | Steers | | | Heifers | | | Calves | |
|  |  |  |  |  |  |  | |  | |  | | |  | | |  | |
|  |  |  |  |  | How often were they sent? | | | During droughts | |  | | | Any time of the year | | |  | |
| What were the benefits of sending animals to a feedlot? Please tick the most appropriate. | | Improved animal health care | |  | Improved animal weight gain |  | | Consistent feed and water availability | |  | | | Accessible market | | |  | |
| What could be the reason for stopping or never sending cattle to the feedlot? | Lack of knowledge |  | Stock theft |  | It is a personal choice. |  | | Unfavorable Policies/Risk | |  | | | Others (specify) | | |  | |
| Which changes would you like to see before participating in feedlots? | | | | Transparent policies | |  | | More awareness workshops | |  | | | | Improved infrastructure | | |  |
| Will you participate in feedlots if such changes can be employed? | | | | Yes | |  | | No | |  | | | | | | | |
| Does sending cattle to feedlots assist in climate change mitigation? | | | | Yes | |  | | No | |  | | Not sure | | |  | | |

1. **Farmers perceptions on role of feedlots as a climate-smart approach to enteric methane**

|  | Yes | No | Not sure |
| --- | --- | --- | --- |
| 1. Have you ever heard of methane? |  |  |  |
| 2. Are you aware of the impact of methane on our environment and livelihoods? |  |  |  |
| 3. If yes, what impacts are you aware of? |  |  |  |
| 4. Did you know that animals also contribute to enteric methane emissions? |  |  |  |
| 5. Which livestock species contributes the most to enteric methane emissions? | Cattle | Goats | Sheep |
| 6. What could be the reason for the above answer in question 5? |  |  |  |
| 7. Can cattle feedlots be considered climate-smart in reducing enteric methane emissions from animals? | Yes | No | Not sure |
| 8. If yes, what could be the reason for the above answer in question 7? |  |  |  |
| 9. Enteric methane is produced in the rumen stomach of cattle through carbohydrate fermentation |  |  |  |
| 10. Does one think cattle production is related to environmental changes? |  |  |  |
| 11. Climate change leads to increased food insecurity |  |  |  |
| 12. Use of feedlots may help reduce enteric methane and improve weight gain |  |  |  |
| 13. Reducing methane output could decrease the chances of drought |  |  |  |
| 14. Feeding concentrates combined with fibre helps reduce methane output |  |  |  |
| 15. The amount of feed consumed by cattle affects methane production |  |  |  |
| 16. Sending or not sending cattle to the feedlot has no difference in weight gain |  |  |  |
